# Three-dimensional computed tomography analysis of the mitral annulus for mitral annuloplasty in 100 cases of robotic mitral valve repair

**DOI:** 10.3389/fcvm.2024.1369801

**Published:** 2024-09-11

**Authors:** Yosuke Takahashi, Akimasa Morisaki, Yoshito Sakon, Kenta Nishiya, Goki Inno, Takumi Kawase, Yukihiro Nishimoto, Munehide Nagao, Noriaki Kishimoto, Kazuki Noda, Toshihiko Shibata

**Affiliations:** Department of Cardiovascular Surgery, Osaka Metropolitan University Postgraduate School of Medicine, Osaka, Japan

**Keywords:** three-dimensional computed tomography, robotic surgery, annuloplasty, mitral valve repair, posterior annular plication rate

## Abstract

**Objectives:**

This study aimed to evaluate the efficacy of preoperative computed tomography in assessing mitral annulus anatomy and the posterior annular plication rate in mitral valve repair with annuloplasty.

**Methods:**

From July 2018 to August 2023, we performed robotic mitral valve repair with ring annuloplasty using a semi-rigid ring in 100 patients. Preoperative anatomical assessment of the mitral annulus was conducted by three-dimensional computed tomography. The ring size was selected based on the perioperative commissure-to-commissure length or the anterior leaflet area.

**Results:**

The mean commissure-to-commissure length, posterior mitral annular length, and minimum distance between the left circumflex artery and mitral annulus values were 31, 109, and 3.8 mm, respectively. No postoperative left circumflex artery injury or ring detachment was recorded. The mean plication rate (length of the posterior side of the prosthetic ring/posterior annular length) was 0.68, and it did not differ among each prosthetic ring size. The posterior plication rate (duplicate ring size 19.4) was a factor influencing the postoperative transmitral mean pressure gradient of 5 mmHg or higher. Freedom from moderate or severe mitral regurgitation was not different between the two groups above and below the posterior plication rate × ring size of 19.4 (*p* = 0.73), with an event-free rate of 97% vs. 96% in 3 years, respectively.

**Conclusions:**

Preoperative evaluation of the mitral annular anatomy is useful for safe mitral valve repair with ring annuloplasty. Determining ring size by focusing on the posterior annular plication rate may be a new method for ring size selection.

## Introduction

Mitral annuloplasty is essential for controlling regurgitation in patients with mitral regurgitation (MR) (1). Mitral annuloplasty for degenerative MR is generally based on ring size selection, as measured by the commissure-to-commissure (Com–Com) length or the anterior leaflet area (2). The usefulness of downsized valvuloplasty has also been reported for ischemic (3) and atrial functional MR (4). These measurements focus on the anterior mitral anatomy.

The mitral annulus is mainly enlarged in the posterior annular direction, and there is no clear data on the extent of posterior annulus plication during annuloplasty. Overplication may occur in cases of excessive dilatation of the posterior annulus, resulting in ring detachment, especially when using semi-rigid or rigid rings (5). Furthermore, excessive plication may elevate the transmitral pressure gradient, causing functional mitral stenosis.

We routinely performed preoperative three-dimensional computed tomography (CT) before surgery for degenerative MR patients to determine the positional relationship between the mitral annulus and the circumflex coronary arteries [left circumflex artery (LCX)]. LCX obstruction is a serious complication when the distance between the LCX and mitral annulus is small, especially when the distance is <3 mm (6). In our routine preoperative analysis, in addition to evaluating the distance between the mitral annulus and LCX, the mitral annular length, Com–Com length, and posterior annular length (PAL) were measured for ring size selection. This study aimed to investigate the effectiveness of mitral anatomy evaluation, posterior annular plication rate, and appropriateness of prosthetic ring size selection in 100 patients with degenerative MR. Furthermore, we investigated how the posterior plication rate affects postoperative echocardiographic parameters.

## Materials and methods

We retrospectively studied 100 consecutive patients with significant (moderate or severe) MR who underwent mitral valve (MV) repair using the da Vinci Xi at Osaka Metropolitan University Hospital from June 2018 to April 2023. All patients had the following: (1) chronic significant MR, (2) chronic heart failure (HF) symptoms of at least New York Heart Association (NYHA) functional class 1, and (3) degenerative or atrial functional MR. We used the loop technique for MV repair and a semi-rigid ring (Carpentier-Edwards Physio Ring II, Edwards Lifesciences, Irvine, CA, USA) for annuloplasty in all 100 patients. Preoperative three-dimensional CT was used to evaluate the anatomy of the MV and the positional relationship between the LCX and annulus, followed by MV surgery on the measurement data. The Institutional Review Board of Osaka Metropolitan University Hospital approved data analysis for this retrospective study and waived the need for patient consent (Approval number: 2020-066).

### Computed tomography scan

**Figure 1:**
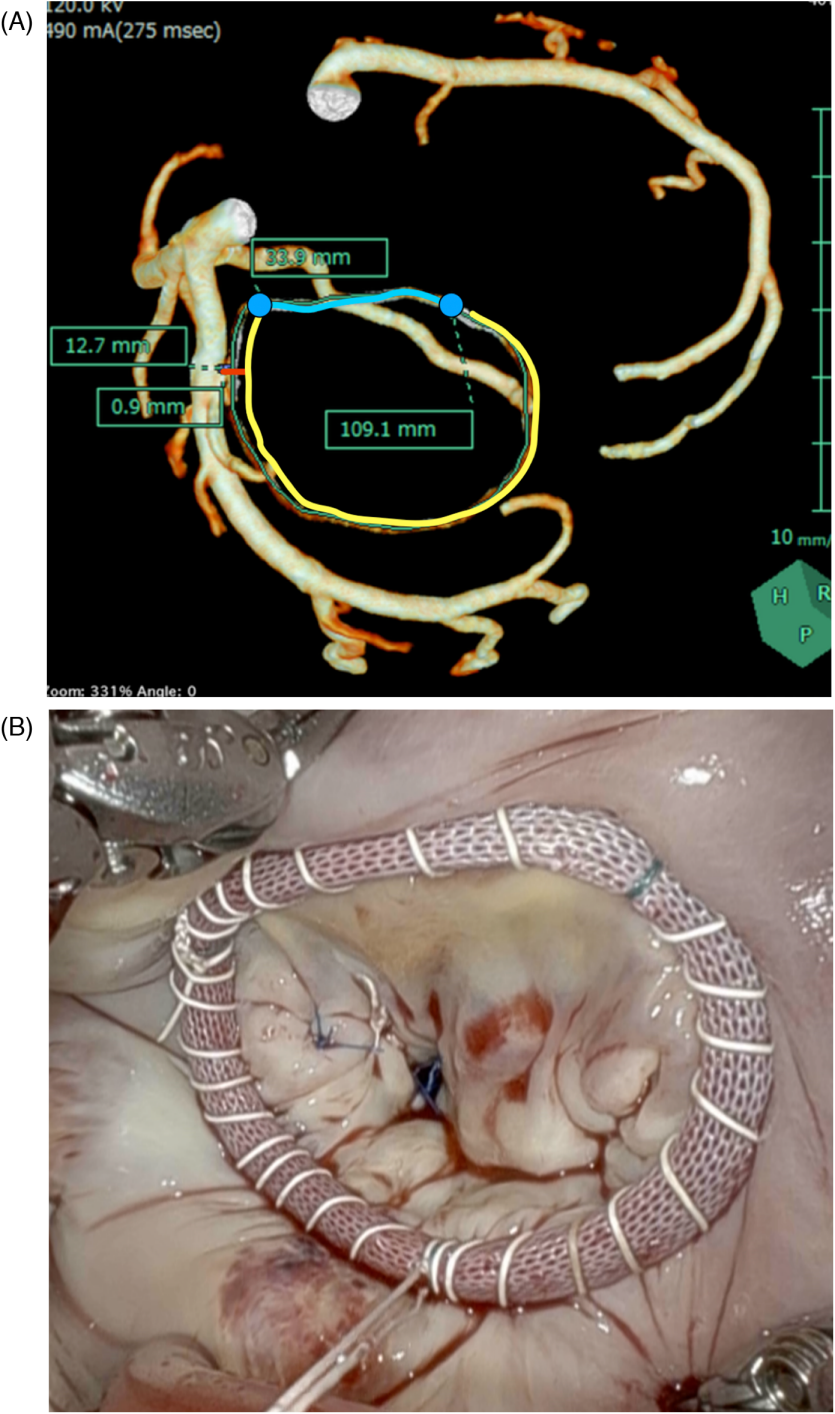
**(A)** The mitral annulus and the coronary artery. mCAD is the shortest distance between the mitral annulus and the LCX artery (red line). The length of mCAD from the edge of the LCX artery to the edge of the mitral annulus was measured. Com–Com is the length between the bilateral fibrous trigone along the anterior annulus (blue line). PAL is the length between the bilateral fibrous trigone along the posterior annulus (yellow line). **(B)** Continuous wrapping sutures for ring annuloplasty using a semi-rigid ring. mCAD, minimum distance between the coronary artery and the mitral annulus; PAL, posterior annular length; Com–Com, commissure-to-commissure.

All patients underwent three-dimensional CT scans before surgery to measure the Com–Com length, PAL, and the minimum distance between the mitral annulus and LCX. A 320-row CT detector (Aquilion ONE/GENESIS Edition, Canon Medical Systems, Otawara, Japan) was used for all scans before surgery of the patients. The CT scan parameters were as follows: 320 rows × 0.5 mm; rotation time, 0.275 s; volume scan; tube voltage, 120 kV; tube current, 800–1,000 mA. Three-dimensional coronary arteries were reconstructed at mid-diastole (75% of the R–R cycle) with a slice thickness of 0.5 mm, a slice interval of 0.25 mm, and a soft reconstruction kernel (FIRST Cardiac Mild). Synapse Vincent software (Synapse Vincent, Fujifilm Medical, Tokyo, Japan) was used to analyze CT data. The coronary arteries were automatically drawn using this software. The mitral annulus was drawn as a three-dimensional line by manually plotting the hinge point of the mitral leaflet. The distance from the mitral annulus to the closest point on the LCX artery wall was measured and expressed as the minimum coronary annular distance (mCAD) (Figure 1A). All image creation and measurements were performed by cardiac surgeons (NK and KNi), after which a radiologist (SI) reassessed the accuracy of the measurements.

### Echocardiography

All patients underwent transthoracic echocardiography (TTE) in our echocardiography laboratory the day before surgery. The MR grade was evaluated by measuring the color Doppler jet area using the Doppler-derived volumetric method or the proximal isovelocity surface area method. For statistical analysis in the present study, the grading of MR or TR was scored as follows: none = 0, none to mild = 0.5, mild = 1, mild to moderate = 1.5, moderate = 2, moderate to severe = 2.5, and severe = 3 (7). The mitral valve area, calculated by pressure half time, was calculated using the following formula: 220 /pressure half time.

### Mitral valve repair using the loop technique

All patients underwent MV repair using the robot. Our loop technique has been described in a previous study (8, 9). The loop set consisted of a felt pledget and CV4 expanded polytetrafluoroethylene (ePTFE) suture loops (Gore-Tex®, WL Gore & Associates, Flagstaff, AZ, USA). The number of loops (usually two or three) depended on the extent of the prolapsed area. The ePTFE suture needles were passed through the corresponding papillary muscle, with the loop set anchored 3–5 mm below the tip of the muscle. The sutures were then passed through the second felt pledget and secured with several knots. In addition, the loop was affixed to the edge of the prolapsing segment with a 5-0 Prolene® (Ethicon, Cincinnati, OH, USA) figure-of-8 suture.

### Mitral annuloplasty

**Figure 2:**
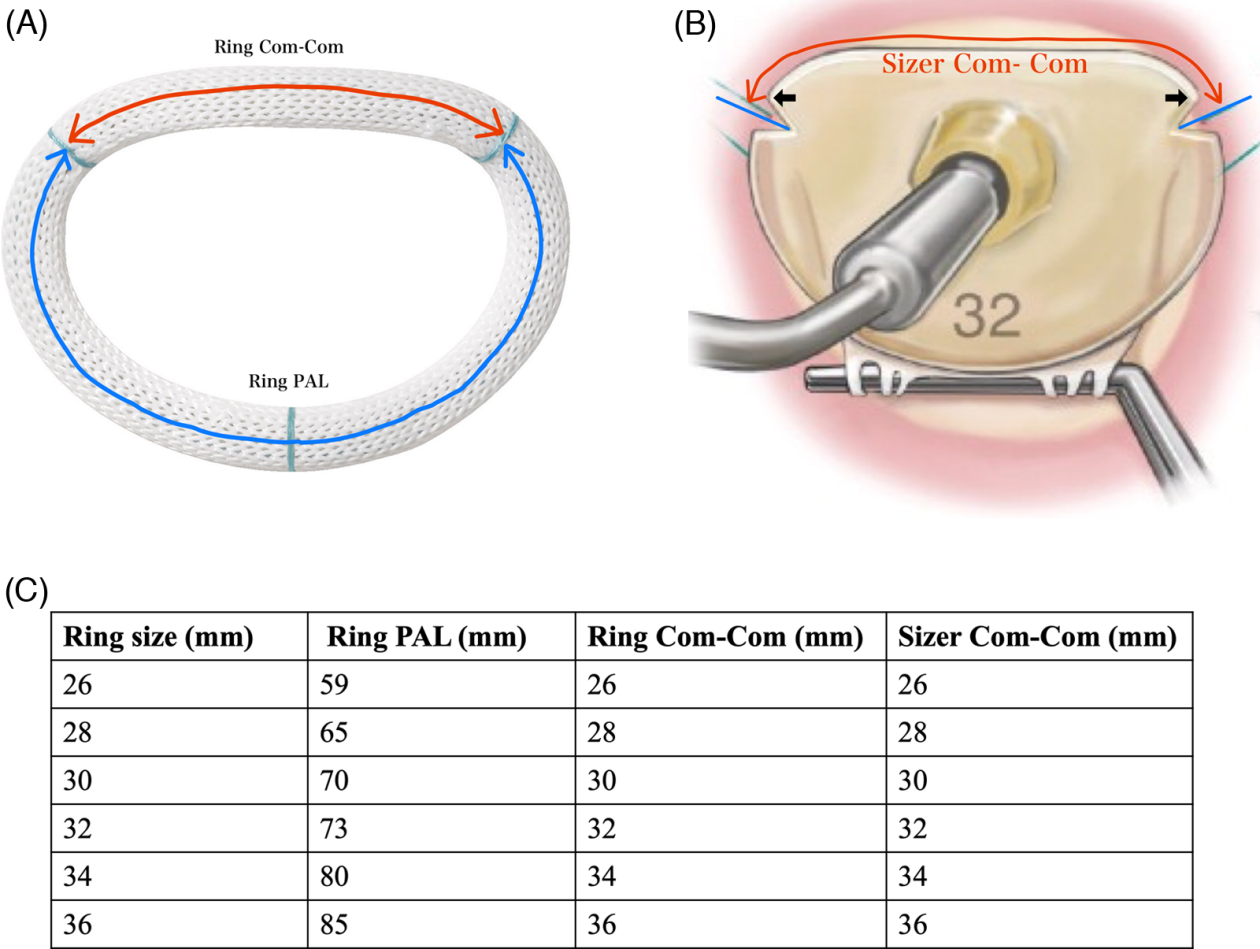
**(A)** Size of equipped company. The ring size was determined based on the Com–Com distance or the anterior leaflet area. The red arrows indicate the distance between the blue lines. The black arrow indicates the location of the bilateral trigons. **(B)** Physio Ring II. The red arrow indicates the ring Com–Com on the anterior annular side. The blue arrow indicates ring length on the posterior annular side. **(C)** Ring PAL, ring Com–Com, and sizer Com–Com for each ring size. PAL, posterior annular length; Com–Com, commissure-to-commissure.

We used the loop technique for MV repair and a semi-rigid ring (Carpentier-Edwards Physio Ring II, Edwards Lifesciences, Irvine, CA, USA) for annuloplasty in all 100 patients. All annuloplasty procedures were performed using the continuous wrapping suture technique (Figure 1B). Ring size was determined based on the Com–Com distance or anterior leaflet area using a sizer (Figure 2A). We placed an interrupted suture at the bilateral fibrous trigons using a 90-cm CV4 PTFE suture (Gore-Tex, Flagstaff, AZ, USA). The threads were sutured continuously at the mitral annulus from the bilateral trigone, effectively wrapping the ring in place. The ring was inserted into a continuous suture without passing through it. In cases where the mCAD was shorter than 3 mm, continuous sutures were performed with a fine pitch and shallow bite.

### Measurement of the plication rate of the posterior mitral annulus and accuracy of ring size selection

The plication ratio of the posterior mitral annulus was expressed as the ratio of the posterior side of the prosthetic ring (ring PAL) to the native PAL. The accuracy of ring size selection was expressed as the ratio of the anterior side of the prosthetic ring (ring Com–Com) to the native Com–Com length. The ring PAL, ring Com–Com (Figure 2B), and size Com–Com (Figure 2A) for each ring size are shown in Figure 2C.

### Follow-up examination and management

Postoperatively, we followed up with the patients through hospital visits, outpatient clinic appointments, and telephone surveys. All patients completed follow-up, which ranged from 90 to 1,608 days (median, 336 days). Postoperative echocardiographic follow-ups were performed at our hospital 7–14 days after surgery and every 3–6 months after discharge. The follow-up duration ranged from 30 to 1,429 days, with a median of 457 days.

### Data collection and statistical analysis

Descriptive statistics for categorical variables were reported as absolute values and percentages, while continuous variables were presented as means and standard deviations. The association between the PAL plication rate and echocardiographic data was analyzed using the Spearman rank correlation coefficient. Receiver operating characteristic (ROC) curves and areas under the curve were assessed using standard methods. The optimal cutoff value was defined as that with the highest sum of sensitivity and specificity. Event-free rates were estimated using the Kaplan–Meier product-limit method. Statistical analysis was performed using R version 3.3.2 (R Foundation for Statistical Computing, Vienna, Austria). Statistical significance was set at *P* < 0.05.

## Results

### Patient profile and preoperative transthoracic echocardiography data

The patient profiles are presented in Table 1. Most patients had hypertension. Almost all patients were MR with degenerative (82%) or Barlow's-like disease (14%). Preoperative TTE showed slightly dilated diastolic dimensions and left atrial volume index (LAVI), while the left ventricular ejection fraction remained within the normal range.

**Table 1:** Patient profile before surgery.

	All patients (*n* = 100)
Age	60 ± 14
Gender (male/female)	72/28
HT	66 (66%)
CRF	5 (5%)
NIDDM	5 (5%)
COPD	5 (5%)
Etiology of MR	
Degenerative	82 (82%)
Barlow's-like	14 (14%)
Rheumatic	1 (1%)
Dilatation of annulus due to AF	3 (3%)
Prolapse site	
Anterior leaflet	15 (15%)
Posterior leaflet	38 (38%)
Bi-leaflet	42 (42%)
Commissure	2 (2%)
Annular dilatation alone	3 (3%)
Preoperative TTE data	
Dd	53 ± 6.8
Ds	32 ± 6.4
EF	61 ± 5.0
LAVI	43 ± 20
TR PG (mmHg)	25 ± 9.2
MR score	2.94 ± 0.19
TR score	1.04 ± 0.52

HT, hypertension; CRF, chronic renal failure; NIDDM, non-insulin-dependent diabetes mellitus; COPD, chronic obstructive pulmonary disease; TTE. Dd, diastolic dimension; Ds, systolic dimension; EF, ejection fraction; LA, left atrium; MR, mitral regurgitation; AF, atrial fibrillation; TR, tricuspid regurgitation; PG, pressure gradient.

### Preoperative computed tomography data

The distribution of PAL across all patients is presented in Table 2. The PAL of most patients ranged from 91 to 130 mm. Patients with Barlow’s disease had longer PAL (120 ± 14 mm) and Com–Com (34 ± 2.8 mm) measurements than degenerative patients (*p* = 0.00114 and *p* = 0.00342, respectively). There were no significant differences in PAL and Com–Com among the prolapse lesion types. In addition, 48% of patients had an mCAD of less than 3 mm and 6% of patients had an mCAD of less than 1 mm. The mCAD was the shortest in the left-dominant type among the three types of coronary systems.

**Table 2:** Preoperative anatomical measurements by three-dimensional computed tomography.

Preoperative computed tomography data	
PAL distribution in all patients (mm)	
70–80	2
81–90	4
91–100	28
101–110	16
111–120	25
121–130	16
131–140	7
141–	2
PAL (mm)	
FED	107 ± 14
Barlow	120 ± 14
Anterior lesion	102 ± 16
Posterior lesion	109 ± 15
Commissure lesion	100 ± 2.0
Bi-leaflet lesion	112 ± 14
Com–Com (mm)	32 ± 3.4
FED	32 ± 3.4
Barlow	34 ± 2.8
Anterior lesion	32 ± 1.9
Posterior lesion	31 ± 3.8
Commissure lesion	30 ± 0.57
Bi-leaflet lesion	33 ± 3.1
Coronary dominance (Co, left, right)	77/13/10
mCAD (mm)	3.36 ± 1.85
mCAD ≤3 (%)	48 (48%)
0–1	6 (6%)
1–2	19 (19%)
2–3	23 (23%)

PAL, posterior annular length; FED, fibroelastic deficiency; Com–Com, commissure-to-commissure; mCAD, minimum distance between the LCX and mitral annulus.

### Perioperative and postoperative data

The perioperative and postoperative echocardiographic data are summarized in Table 3. One-fourth of the patients underwent concomitant procedures, and six patients (6%) experienced systolic anterior motion. Of the six patients with systolic anterior motion, two required additional height reduction under the second pump run, resulting in the recovery of systolic anterior movement. Other four patients recovered from systolic anterior movement through volume loading. The plication rate of the PAL was 0.68 ± 0.069 and did not differ depending on each ring size. Based on preoperative Com–Com measurements, the accuracy of the selected ring size was 0.99 ± 0.08. There were neither perioperative deaths nor cases of low cardiac output syndrome. One patient required re-exploratory thoracotomy due to bleeding. The MR scores at both postoperative periods and the most recent periods were 0.49 ± 0.47 and 0.89 ± 0.67, respectively. No patient required reoperation. Two patients had moderate or severe MR, and there were no instances of recorded ring detachment or LCX obstruction.

**Table 3:** Perioperative data.

	All (*n* = 100)
Operative data (*n* = 100)	
Mitral valve ring size	32 ± 2.8
Commissural edge to edge	0.83 ± 0.9
Total number of neochords	4.0 ± 1.6
Concomitant procedures	
TAP	22 (22%)
MAZE	20 (20%)
LAA closure	25 (25%)
SAM	6 (6.0%)
Operation time (min)	321 ± 79
Cross clamp time (min)	143 ± 37
Ring size/Com–Com	0.99 ± 0.08 (%)
Plication rate of PAL (%) (mm)	68 ± 6.9 (%)
26 (*n* = 4)	69 ± 8.9
28 (*n* = 15)	68 ± 7.4
30 (*n* = 24)	66 ± 5.7
32 (*n* = 24)	68 ± 9.3
34 (*n* = 22)	70 ± 6.1
36 (*n* = 11)	67 ± 4.0
Postoperative echocardiography	(*n* = 100)
Dd	48 ± 6.4
Ds	31 ± 6.5
EF	55 ± 7.0
LAVI	43 ± 20
TRPG	25 ± 9.3
MR score	0.49 ± 0.47
TR score	0.82 ± 0.38
Mean PG	2.3 ± 0.97
Most recent echocardiography	(*n* = 100)
Dd	45 ± 5.3
Ds	28 ± 5.4
EF	58 ± 5.1
LAVI	25 ± 10
TRPG	19 ± 5.6
MR score	0.89 ± 0.67
TR score	0.80 ± 0.33
Mean PG	2.9 ± 1.2

TAP, tricuspid annuloplasty; LAA, left atrial appendage; SAM, systolic anterior motion; Com–Com, commissure-to-commissure; PAL, posterior annular length, Dd, diastolic dimension; Ds systolic dimension; EF, ejection fraction; LAVI, left atrium volume index; TR, tricuspid regurgitation; PG, pressure gradient; MR, mitral regurgitation; TRPG, tricuspid regurgitation pressure gradient.

### Posterior plication rate × ring size as a factor influencing postoperative transmitral mean pressure gradient

**Figure 3:**
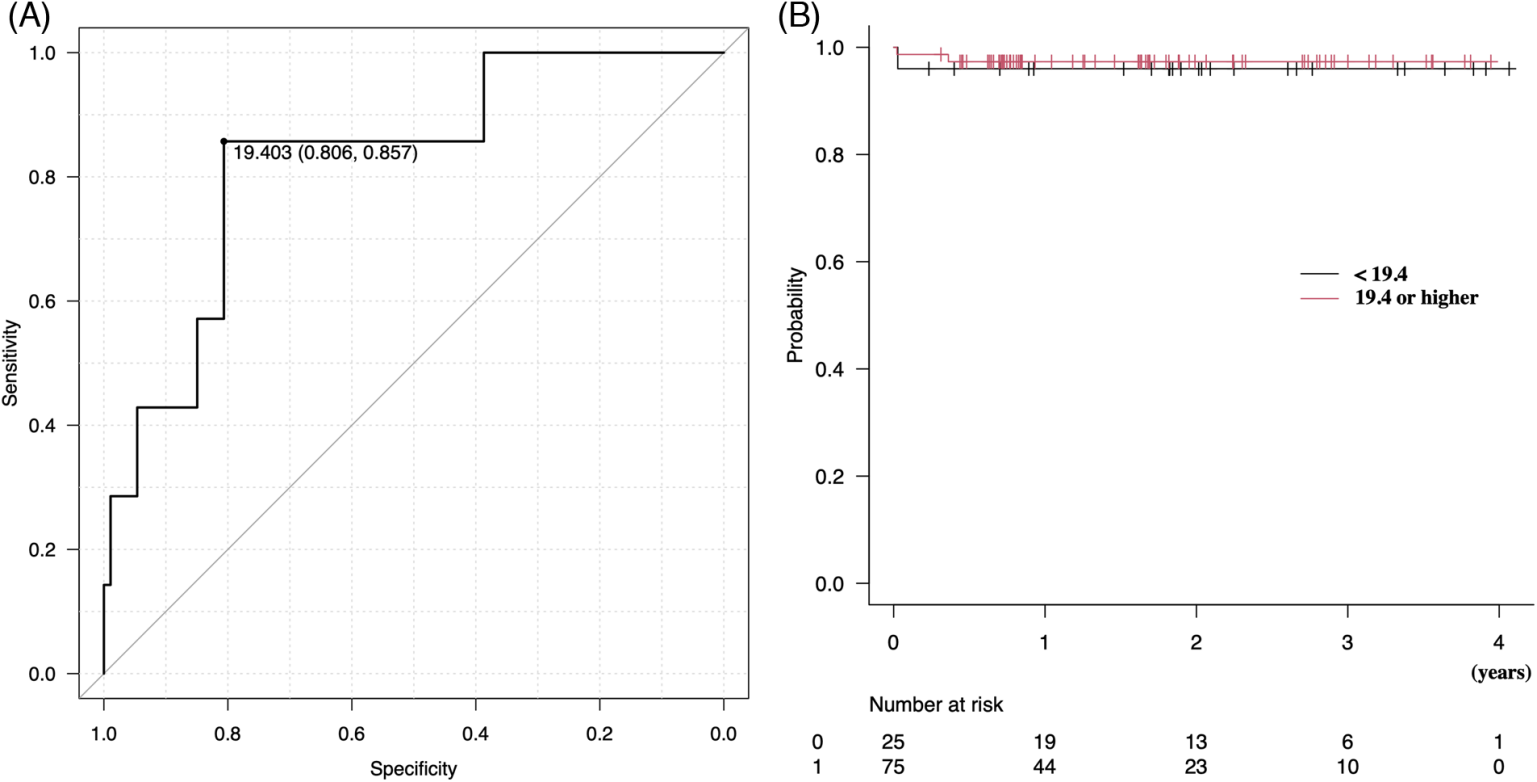
**(A)** Receiver operating characteristic curves were used to evaluate the optimal cutoff value of posterior plication rate × ring size for a postoperative transmitral mean pressure gradient of 5 mmHg or higher. **(B)** Kaplan–Meier analysis of freedom from above moderate or severe MR. MR, mitral regurgitation.

ROCs were used to evaluate the optimal cutoff value of plication rate × ring size for determining a postoperative mean transmitral mean pressure gradient of 5 mmHg or more (Figure 3A). A PAL × ring size of 19.4 provided the maximum sum of sensitivity and specificity for predicting the postoperative mean transmitral pressure gradient (81% and 86%, respectively). The area under the curve was 0.83. The plication rate × ring size corrected by body surface area (BSA) showed a cutoff value of 11.1, with the maximum sensitivity and specificity for predicting the postoperative mean transmitral pressure gradient of 82% and 86%, respectively. The area under the curve was 0.82.

### Mean transmitral pressure gradient for each ring size and each plication rate

Mean transmitral pressure gradient for each ring size and each plication rate are shown in Table 4.

For smaller ring sizes (26 and 28 mm), the mean transmitral pressure gradient was high when the posterior plication rate was less than 0.65. On the other hand, for ring sizes larger than 34 mm, the mean transmitral pressure gradient remained low even when the suture ratio was less than 0.6.

**Table 4:** Mean transmitral pressure gradient for each ring size and each plication rate.

	Transmitral mean PG (mmHg)			MVA (cm^2^)		
Ring PAL/PAL	Ring size					
	26 and 28 mm	30 and 32 mm	34 and 36 mm	26 and 28 mm	30 and 32 mm	34 and 36 mm
<0.59	4.3 (*n* = 3)	3.7 (*n* = 7)	1.0 (*n* = 2)	2.3 (*n* = 3)	2.5 (*n* = 7)	2.0 (*n* = 2)
0.60–0.64	4.0 (*n* = 1)	2.5 (*n* = 14)	2.0 (*n* = 9)	2.2 (*n* = 1)	2.5 (*n* = 14)	2.6 (*n* = 9)
0.65–0.69	3.6 (*n* = 8)	2.0 (*n* = 10)	2.4 (*n* = 7)	2.3 (*n* = 8)	2.3 (*n* = 10)	2.5 (*n* = 7)
0.70 ≦	3.0 (*n* = 7)	2.4 (*n* = 19)	1.9 (*n* = 13)	2.2 (*n* = 7)	2.4 (*n* = 19)	2.5 (*n* = 13)
[(Ring PAL/PAL) × ring size]	Transmitral mean PG (mmHg)			MVA (cm^2^)		
<19	3.5 ± 1.8 (*n* = 15)			2.4 (*n* = 15)		
19–21	2.8 ± 1.1 (*n* = 32)			2.3 (*n* = 32)		
21–23	2.5 ± 1.2 (*n* = 23)			2.5 (*n* = 23)		
23<	2.2 ± 0.8 (*n* = 30)			2.5 (*n* = 30)		

PAL, posterior annular length; PG, pressure gradient; MVA, mitral valve orifice area.

As for the (ring PAL/PAL) × ring size, the mean transmitral pressure gradient was higher (3.5 ± 1.8 mm Hg) when the (ring PAL/PAL) × ring size was less than 19.0.

### Freedom from recurrence of mitral regurgitation

Figure 3B shows the Kaplan–Meier analysis, which demonstrated that freedom from moderate or severe MR was not different between the two groups above and below the posterior plication rate × ring size of 19.3 (*p* = 0.73), with event-free rates of 97% vs. 96% at 1 year and 97% vs. 96% at 3 years, respectively. Differences in the PAL plication rate did not affect MR recurrence in the mid-term periods.

### Effect of the plication of posterior mitral annular length on postoperative cardiac function

**Figure 4:**
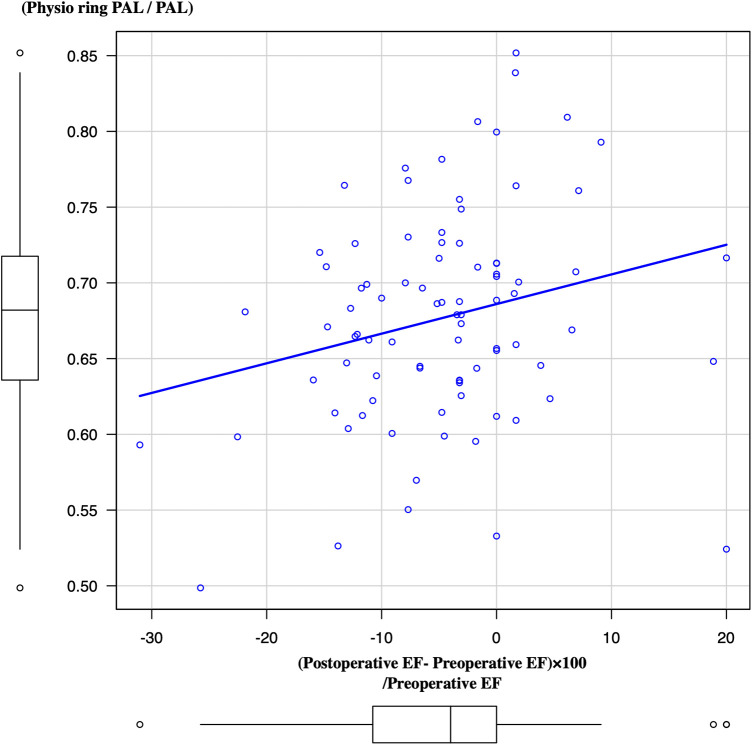
Association between the plication rate of PAL and LV ejection fraction from preoperative periods to most recent periods. PAL, posterior annular length; LV, left ventricle.

Lower PAL plication resulted in a higher recovery rate of EF during the most recent periods (*p* = 0.0268) (Figure 4).

## Discussion

Our data showed the following findings: (1) the mean plication rate of PAL was 0.68 and did not differ depending on the ring size; (2) preoperative CT evaluation of mitral annulus anatomy was effective for mitral ring size selection and the prevention of LCX injury; (3) a posterior plication rate × ring size 19.4 was a factor influencing postoperative transmitral mean pressure gradient of 5 mmHg or higher; and (4) a posterior plication rate × ring size did not affect MR recurrence in the mid-term periods.

Regarding ring size selection, measuring the Com–Com distance or anterior leaflet area without downsizing is the gold standard for degenerative MR (2, 10, 11). In contrast, Murashita et al. reported restrictive annuloplasty for degenerative MR (12), where a 63-mm flexible band was used in all cases. In light of our results, plication to 63 mm is similar to that of the 28-mm Physio Ring II. We believe that some patients may develop complications with mitral stenosis after ring annuloplasty due to overplication. Based on our results, determining the ring size by focusing on the posterior annular plication rate is valid in terms of over- or underplication, which might be a new concept of ring size selection. Further studies are needed to clarify the effectiveness of determining the ring size using the posterior leaflet plication rate.

The accuracy of preoperative Com–Com measurements in predicting ring size was 0.99 when we used the semi-rigid ring (Carpentier-Edwards Physio Ring II, Edwards Lifesciences, Irvine, CA, USA) in this study. This means that preoperative CT measurements were consistent with the intraoperative size measurements. Notably, the plication rate of the PAL (length of the posterior side of the prosthetic ring/PAL) was approximately 0.68, which was almost the same for each prosthetic ring size. We hope that our data can serve as a guide for selecting the ring size when encountering cases with more or fewer plications based on a PAL plication ratio of 0.68.

LCX injury is a matter of concern when performing annuloplasty on the posterior annular side. Therefore, we preoperatively evaluated the minimum distance and its point between the LCX and the mitral annulus. Notably, 48% of patients had an mCAD of less than 3 mm. Our study demonstrated that the mCAD was very short in patients with the left-dominant type and AF. In cases where the distance between the mitral annulus and LCX is less than 3 mm, we try to perform the wrapping suture around the LCX with shallow stitches, as many surgeons consider that suturing perpendicular to the mitral annulus increases the risk of LCX injury. However, this study showed that it can be performed safely by performing preoperative CT measurements. To date, no study has measured mCAD or provided recommendations for caution during mitral annuloplasty. We hope that preoperative measurement of the anatomic positioning of the coronary arteries and mitral annulus will be essential for safe annuloplasty.

Several reports have described mitral stenosis after mitral valve repair (10–14). Kawamoto et al. reported that an elevated transmitral mean pressure gradient led to an increased transtricuspid pressure gradient, progressive TR, and new-onset atrial fibrillation (10). Many researchers have defined mitral stenosis as a mitral valve area <1.5 cm^2^ or a transmitral mean pressure gradient of 5 mmHg, which can cause heart failure symptoms and reduce freedom from cardiovascular events (13–15). In our data, seven patients developed a transmitral mean pressure gradient of 5 mmHg or more in the mid-term periods. As the effect of the plication rate on the mean pressure gradient varies depending on the ring size, we propose an index that multiplies the ring size by the plication rate. The PAL × ring size of 19.4 was identified as the cutoff value for predicting a postsurgery mitral pressure gradient of 5 mmHg or more. Based on our results, care should be taken to avoid overplication for cases where the mean transmitral PG was 3.7 mmHg with a plication rate less than 0.60 and rings smaller than 32 mm (plication rate × ring size: 19.2), and additional techniques such as commissural edge-to-edge and cleft suture should also be carefully used during mitral valve repair.

Annular dilatation may affect the posterior annular side (16). El-Tallawi et al. reported that leaflet remodeling occurred in patients with primary and secondary MR patients (17). In patients with secondary MR, the balance between leaflet and left ventricle (LV) remodeling is key to determining the progression of MR severity (18). In poor leaflet remodeling cases, overplication was necessary to decrease MR. On the other hand, in patients with Barlow's disease, forme fruste, or sufficient leaflet remodeling cases, the leaflet area or volume was sufficient for good coaptation. From our results, cases of a markedly enlarged posterior annulus compared to the anterior annulus, requiring excessive posterior annular plication, might result in less recovery of LV systolic function after surgery than those in other patients. We believe that PAL plication <0.6 might not be necessary for such patients.

Preoperative coronary artery examination is always necessary for mitral valve surgery. We routinely performed three-dimensional CT, including cardiac CT, to evaluate the coronary artery. Coronary angiography is only performed when coronary stenosis is suspected based on cardiac CT findings. As most patients undergoing mitral valve repair are relatively young with little atherosclerosis, the coronary arteries are assessed by cardiac CT without major problems.

We previously reported the results of 351 cases of mitral valve repair performed under median sternotomy or right mini-thoracotomy (19). In our report, coronary artery bypass surgery was necessary due to LCX injury during mitral annuloplasty in two cases: one case occurred during a median sternotomy, while the other occurred during a right mini-thoracotomy (19). We have also encountered three left ventricular ruptures, one of which involved a myocardial infarction in the LCX region, resulting in a left ventricular plasty on postoperative day 9 (19). This experience led to preoperative three-dimensional cardiac CT scans to measure the distance between the LCX and the mitral annulus. Furthermore, CT data are used to determine not only the positional relationship between the mitral annulus and the coronary arteries but also to measure mitral annulus anatomy and evaluate the distribution of the papillary muscles. In addition, using Vincent (Fuji Film, Tokyo, Japan), we surgeons can construct the images ourselves in about 15 min. CT analysis with Vincent is also widely used for preoperative CT analysis in other fields, e.g., liver surgery. The ability to obtain so much information in a single three-dimensional contrast-enhanced CT, as described above, is vital from a cost-effectiveness perspective. Three-dimensional transesophageal echocardiography (TEE) is an alternative to cardiac CT. With three-dimensional TEE, mitral annulus anatomy can also be measured, and furthermore, both systolic and diastolic data can be easily obtained. However, coronary angiography is required for coronary assessment. As we also look at the utility of three-dimensional TEE, further study is necessary to evaluate its accuracy by comparing three-dimensional CT and three-dimensional TEE data.

### Limitations

This study has several limitations. First, our study had a retrospective design, which included only a small number of patients. Nevertheless, our data were obtained from a single reference surgical team committed to attempting valve repair in almost all cases of degenerative prolapse. Furthermore, all surgeries were performed by the first author (YT) using a robot with an excellent operative view. Second, measurement bias in preoperative CT measurements and intraoperative measurements may occur. Third, we performed postoperative enhanced three-dimensional CT only in patients whose postoperative creatine kinase-MB (CK-MB) levels were >100 IU. Fortunately, no patients with coronary obstruction underwent postoperative enhanced three-dimensional CT. Fourth, it would be important to compare the wrapping suture technique with a semi-rigid ring not only to other types of technique including interrupted suturing technique but also to other types of rings. Fifth, if the MV orifice area, calculated by the continuity equation, was determined using the stroke volume measured in the LV outflow tract divided by the integral of the trans mitral valve velocity during diastole, the relationship between the posterior plication ratio and the effective valve orifice area may be more accurately calculated (20). As this was a retrospective study, only pressure half time was available, but it would be desirable to measure MV area in future studies.

Finally, we did not compare the plication rate using semi-rigid rings with that using other ring types. Although the mitral repair technique employed was the loop technique without leaflet resection in all degenerative cases, the number of loops and additional techniques were not consistent.

## Conclusion

Preoperative evaluation of mitral annular anatomy is useful for safe ring annuloplasty. Determining ring size by focusing on the posterior annular plication rate may be a new concept for ring size selection.

## Data Availability

The raw data supporting the conclusions of this article will be made available by the authors without undue reservation.
